# Functional Neurological Disorder Presenting as Psychogenic Non-epileptic Seizures Associated With Recurrent Hemoptysis: A Diagnostic Challenge

**DOI:** 10.7759/cureus.114794

**Published:** 2026-08-19

**Authors:** Rithika Ramadugu, Chandra Varshini Pidikiti, Iliana Garrido Flores, Alesha Cordero Peña, Angelis Almonte, Jose L Gonzalez, Abigail Almonte, Tarun Kumar Suvvari

**Affiliations:** 1 General Practice, TGSRTC Hospital, Hyderabad, IND; 2 Gastroenterology, Kamineni Academy of Medical Sciences and Research Centre, Hyderabad, IND; 3 Primary Health Care, National Health Service (SNS), Santo Domingo, DOM; 4 Medicine, Instituto Tecnológico de Santo Domingo, Santo Domingo, DOM; 5 General Medicine, National Health Service (SNS), Santo Domingo, DOM; 6 Medicine, Universidad Nacional Pedro Henríquez Ureña, Santo Domingo, DOM; 7 General Medicine, Rangaraya Medical College, Kakinada, IND; 8 Research, Squad Medicine and Research (SMR), Kakinada, IND

**Keywords:** adolescent, case report, conversion disorder, functional neurological disorder, hemoptysis, psychogenic non-epileptic seizures

## Abstract

Functional neurological disorder (FND), previously termed conversion disorder, is characterized by neurological symptoms that are incongruent with recognized neurological or medical conditions. Psychogenic non-epileptic seizures (PNES) are among its most common manifestations; however, hemoptysis occurring in association with PNES is exceedingly rare. A 15-year-old male presented with recurrent seizure-like episodes of one-year duration that progressively increased in frequency. During episodes, he remained conscious, responsive, and able to communicate, with no post-ictal confusion, urinary incontinence, or tongue biting. The events were accompanied by recurrent hemoptysis, chest pain, and hyperhidrosis. Extensive investigations, including hematological and biochemical testing, electroencephalography, magnetic resonance imaging of the brain, autoimmune encephalitis panel, upper gastrointestinal endoscopy, computed tomography of the chest, bronchoscopy, and otorhinolaryngological evaluation, failed to identify an organic etiology. Following multidisciplinary assessment by neurology and psychiatry teams, a diagnosis of FND presenting as PNES with associated hemoptysis was established. The patient was managed with psychotherapy, psychiatric support, selective serotonin reuptake inhibitors (SSRIs), and clonazepam, with tranexamic acid administered during episodes of hemoptysis, resulting in symptomatic improvement. This case highlights an unusual presentation of FND and emphasizes the importance of comprehensive multidisciplinary evaluation in patients with PNES and unexplained hemoptysis. Recognition of this rare association may prevent unnecessary investigations, facilitate timely diagnosis, and enable appropriate psychological and supportive management.

## Introduction

Functional neurological disorder (FND), previously known as conversion disorder, is characterized by neurological symptoms that are incongruent with recognized neurological or medical conditions and cannot be fully explained by structural or pathophysiological abnormalities [1]. This disorder, interfacing between neurology and psychiatry, presents with symptoms including blindness, paralysis, dystonia, psychogenic nonepileptic seizures, dysphagia, anaesthesia, tics involving the motor system, difficulty walking, hallucinations, and dementia [2]. It is a common cause of neurological disability, accounting for a substantial proportion of outpatient neurology consultations and emergency presentations [3]. With a reported incidence of 4-12 per 100,000, it is the second most common diagnosis in neurology clinics [1]. The disorder is thought to arise from complex interactions among biological, psychological, and social factors, often resulting in involuntary disturbances of motor, sensory, cognitive, or behavioural functioning [2,4].

Psychogenic non-epileptic seizures (PNES) represent one of the most frequent manifestations of FND and are characterized by paroxysmal episodes resembling epileptic seizures in the absence of corresponding epileptiform activity on electroencephalography [5]. Distinguishing PNES from epileptic seizures remains clinically challenging and frequently requires detailed clinical assessment, video-electroencephalographic evaluation, and exclusion of alternative neurological diagnoses [6]. Delayed recognition may result in repeated hospitalizations, unnecessary investigations, and inappropriate treatment [5,7]. Although PNES may present with a broad spectrum of associated symptoms, hemoptysis has rarely been described in this setting and may prompt extensive evaluation for pulmonary, gastrointestinal, hematological, or factitious causes [8,9]. The occurrence of recurrent hemoptysis during seizure-like episodes poses a significant diagnostic challenge and may obscure the underlying functional nature of the disorder. We report an unusual case of FND presenting with PNES accompanied by recurrent hemoptysis in an adolescent male.

## Case presentation

A 15-year-old Indian male patient presented to the general OPD through a referral with episodes of seizure-like activity for the past year. It started with one to two episodes every week and gradually progressed, presenting with multiple episodes each day, during which he used to be conscious and talk to people around him. The symptoms varied from mild tremors to seizure-like activity. The episodes involved stiffening, causing the patient to lose balance, accompanied by jerking movements of all four limbs and sometimes the head. The episodes were accompanied by bleeding through the mouth, chest pain, and hyperhidrosis. No history of voiding or vomiting during the episode, and no post-ictal state was noted. The patient has had asthma for seven years, which is well under control, with the use of salbutamol inhalers, as needed. The patient reported awareness during the episodes, which lasted between 5 minutes and 20 minutes (longest duration), and could communicate when one was about to start, appears to increase during socialization, even with parents or medical personnel, but sometimes even when isolated. The pseudoseizures were almost always accompanied by diffuse chest pain (which he reported felt like a squeezing type) and hyperhidrosis during the episode, but hemoptysis usually happened during episodes longer than 10 minutes.

Upon examination, the patient was conscious and oriented to time, place, and person. General physical exam did not yield any significant findings. The vital signs were normal. During the seizure-like activity, the patient was able to follow instructions and communicate verbally with effort. Neurological examination was unremarkable, with no focal deficits. The patient was admitted for further workup and to rule out factitious disorder in the ICU. The patient was provisionally diagnosed with psychogenic non-epileptic seizures (PNES). Upon further questioning, no stressful event was reported prior to the sudden onset of the symptoms, a year ago. The parents and family report no traumatic event or psychiatric symptoms prior. The patient was not on any medication, except for asthma, and no substance abuse was noted either. No mental health issues or neurological issues were reported among the family. The patient shares a strong bond with both his parents and a competitive relationship with his younger brother and a few close friends. As a child, he reportedly did not have any developmental delays, as verified by previous doctors' notes. The patient was in ninth grade when the symptoms appeared; he struggled but got through boards and is in preparatory school for professional education. Prior to the onset of FND, he was doing great, with multiple friends in school; later, he has struggled to hold friendships due to the stigma and has struggled with education, as the exam stress sometimes precipitates the episodes. The relevant investigations, such as complete blood count (CBC), bleeding time (BT), clotting time (CT), prothrombin time (PT), serum electrolytes, etc., were within normal limits. IgE levels were elevated (2,251 IU/mL), which could be suggestive of asthma. Further investigations done were within normal ranges. Electroencephalogram (EEG) done in awake and asleep states revealed a borderline EEG, suggestive of pseudo seizures. Sharp waves were noted.

**Figure 1:**
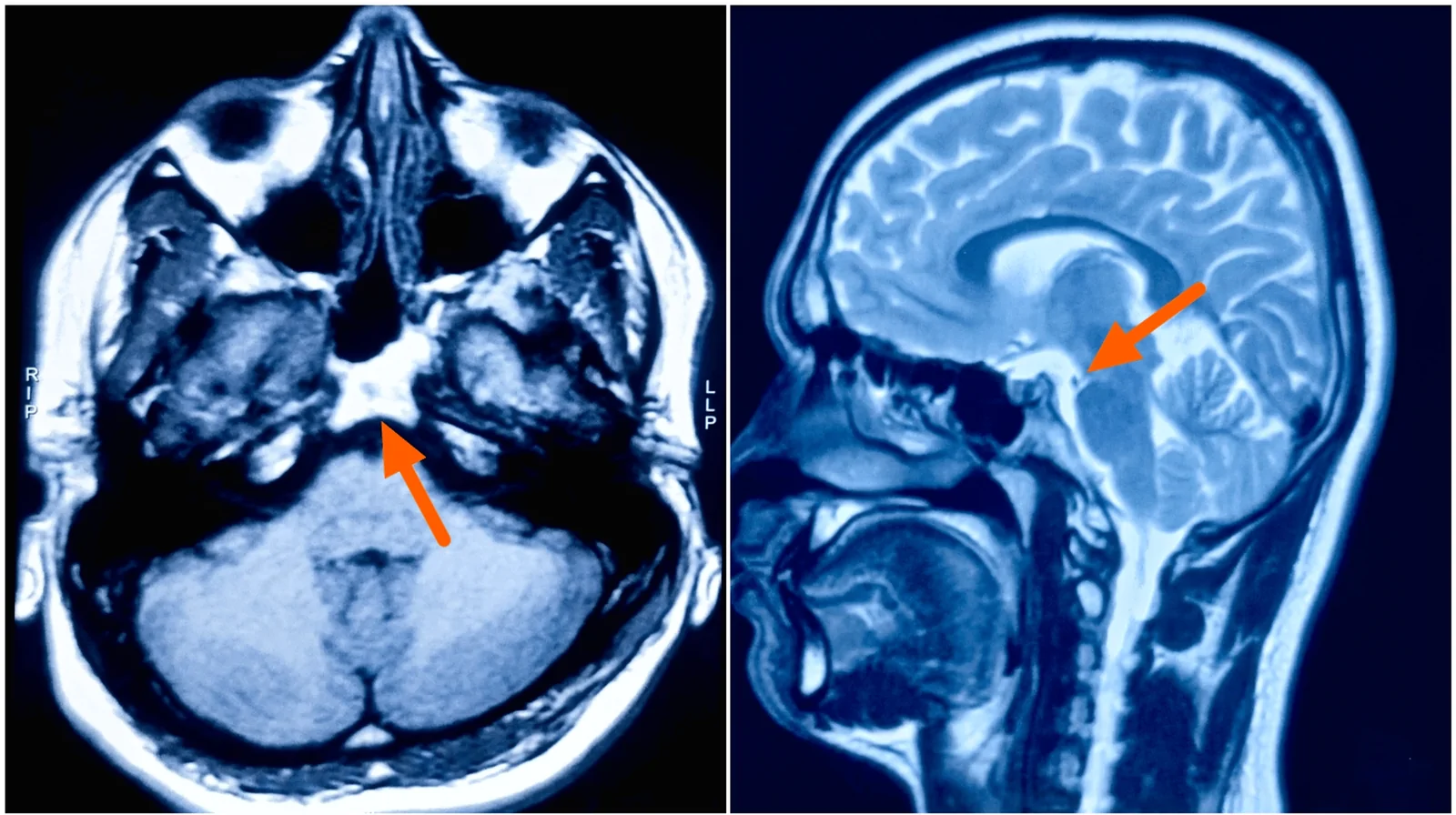
Brain MRI T2-weighted image demonstrating a 4.5 × 5 mm hypointense nodule in the intermediate zone of the pituitary gland (arrows), suggestive of a Rathke's cleft cyst with calcification

Magnetic resonance imaging (MRI) brain revealed a 4.5 x 5 mm T2 hypointense nodule in the intermediate zone of the pituitary gland, which was suggestive of Rathke's cleft cyst/calcification (Figure 1).

Serum prolactin, thyroid-stimulating hormone (TSH), and serum cortisol were evaluated to assess pituitary hormonal function and exclude endocrine abnormalities and pituitary metaplasia. The autoimmune encephalitis panel consisting of N-methyl-D-aspartate (NMDA) receptor, α-amino-3-hydroxy-5-methyl-4-isoxazolepropionic acid receptor subunit 1 (AMPA-GluR1), γ-aminobutyric acid type B (GABA-B) receptor antibody, etc. was negative. Upper GI endoscopy did not reveal any bleeding spots or any other significant findings. Contrast-enhanced CT (CECT) chest revealed mild cylindrical changes in both lungs, following which a bronchoscopy was done, and no bronchiectasis was noted. X-ray of the paranasal sinuses and otorhinolaryngology (ENT) consultation were taken to rule out any ENT pathologies. Ophthalmology consultation was taken to exclude any vision disturbances. A formal mental status and psychometric assessment were not conducted as part of this workup. Neurology and psychiatry consultation were taken, and the patient was diagnosed with FND (conversion disorder) presenting with psychogenic nonepileptic seizures and hemoptysis. The diagnosis was established on the basis of a comprehensive multidisciplinary evaluation that excluded organic neurological, cardiopulmonary, gastrointestinal, endocrine, autoimmune, and ENT/ophthalmological causes, combined with positive clinical features supporting a functional etiology-retained awareness and verbal interaction during episodes, situational triggering by social presence and academic stress, and synchronous four-limb jerking (Table 1). Uncontrolled asthma was considered a differential diagnosis, considering overlapping autonomic symptoms, but was excluded because the patient's asthma was well-controlled on as-needed salbutamol with no correlating respiratory findings during episodes.

**Table 1:** Summary of the diagnostic investigations and results AMPA-GluR1: α-amino-3-hydroxy-5-methyl-4-isoxazolepropionic acid receptor subunit 1; CECT: contrast-enhanced CT; GABA-B: γ-aminobutyric acid type B; NMDA: N-methyl-D-aspartate; TSH: thyroid-stimulating hormone;

Investigation	Result
Complete blood count (CBC)	Hb: 14.4 g/dL; TLC: 9,200/µL; platelets: 280,000/µL
Bleeding time	2 min 30 sec
Clotting time	9 min 47 sec
Prothrombin time (PT)	13.3 sec
Serum electrolytes	Na⁺ 142 mmol/L; K⁺ 4.0 mmol/L; Cl⁻ 101 mmol/L
Serum IgE	2,251 IU/mL (elevated)
Serum prolactin	17 ng/mL
Serum phosphorus	4.75 mg/dL
TSH	3.442 µIU/mL
Serum cortisol	17 µg/dL
2D echocardiography	Normal study; EF 70%
Autoimmune encephalitis panel (NMDA, AMPA-GluR1, GABA-B)	Negative
EEG (awake and asleep)	Borderline; sharp waves noted
MRI brain	4.5 × 5 mm T2-hypointense pituitary nodule, suggestive of Rathke's cleft cyst/calcification
Upper GI endoscopy	No bleeding source or other significant findings
CECT chest	Mild cylindrical changes in both lungs
Bronchoscopy	No bronchiectasis
X-ray paranasal sinuses	Normal

Following the diagnosis, the patient was managed with psychotherapy and psychiatric support. Cognitive behavioral therapy (CBT) was conducted at 15-day intervals for the first year, subsequently continuing at eight-week intervals in line with psychiatric follow-up. Pharmacological treatment included escitalopram 10 mg in the morning and clonazepam 0.5 mg once daily at bedtime (HS), while tranexamic acid 500 mg, administered orally on an as-needed (SOS) basis, was given during episodes of hemoptysis. No adverse effects were noted with pharmacotherapy. The patient and caregivers were counseled regarding the diagnosis and advised regular follow-up. He was discharged on day six in stable condition, continued on escitalopram 10 mg in the morning, clonazepam 0.5 mg HS, and tranexamic acid 500 mg orally SOS, with outpatient psychiatric follow-up arranged. Improvement in symptom frequency and severity was observed approximately four to six weeks after initiation of psychotherapy and pharmacotherapy. At the most recent follow-up, the patient continues on the same pharmacological regimen with follow-up visits every eight weeks. He reports a significant reduction in frequency and severity of episodes and an improved quality of life compared to baseline. The chronological progression of symptoms, treatment initiation, response, and subsequent follow-up is summarized in Table 2.

**Table 2:** Case timeline

Period	Event
~1 year before presentation	Onset, 1-2 episodes/week
Progressive course	Multiple episodes/day
Presentation, June 2023	Full multidisciplinary workup, ICU admission
Day 6	Discharged on escitalopram 10 mg AM, clonazepam 0.5 mg HS, tranexamic acid 500 mg SOS; CBT every 15 days
4-6 weeks post-treatment	Reduction in episode frequency/severity noted
1 year post-diagnosis onward	CBT shifted to every 8 weeks, aligned with psychiatric follow-up
July 2026	Current status: doing well, improved quality of life

## Discussion

FND, previously known as conversion disorder, is characterized by neurological symptoms that are incongruent with recognized neurological or medical conditions and cannot be fully explained by structural pathology [1,2,4]. Although traditionally regarded as a diagnosis of exclusion, contemporary diagnostic frameworks emphasize the identification of positive clinical features alongside appropriate exclusion of alternative neurological disorders [7-9]. FND is an important cause of neurological disability and commonly presents with motor, sensory, cognitive, or seizure-like symptoms. PNES represent one of its most frequent manifestations and often pose significant diagnostic challenges because of their close resemblance to epileptic seizures [7,10,11].

In the present case, several clinical features favoured PNES over epileptic seizures. The patient remained conscious and responsive during episodes, was able to communicate with surrounding individuals, lacked post-ictal confusion, and demonstrated variable motor manifestations. Contemporary diagnostic frameworks for FND increasingly rely on such positive semiological features rather than exclusion alone - including retained awareness, situational or socially triggered onset, asynchronous or variable motor activity, and fluctuating course - which were all present in this case and support a positive diagnosis of PNES rather than one reached solely by ruling out epilepsy [9]. Furthermore, electroencephalography did not reveal findings consistent with epilepsy. The chest pain and hyperhidrosis accompanying this patient's episodes are consistent with the autonomic component reported in PNES; prior work has found PNES patients over-report ictal autonomic symptoms such as palpitations and diaphoresis relative to objectively recorded physiological change, and preictal increases in respiratory rate and heart rate have been observed, supporting a role for autonomic dysregulation in PNES pathophysiology where PNES patients showed over-reporting of ictal symptoms such as palpitations and diaphoresis disproportionate to their recorded heart rate, consistent with impaired interoceptive processing as part of a functional disorder spectrum. These observations, together with extensive neurological evaluation, supported the diagnosis of PNES. Distinguishing PNES from epileptic seizures is clinically important, as misdiagnosis may expose patients to unnecessary investigations and prolonged treatment with antiseizure medications [12-14]. Careful assessment of seizure semiology, consciousness during episodes, post-ictal features, and electroencephalographic findings remains essential in establishing the diagnosis [15,16].

The most unusual aspect of this case was the occurrence of recurrent hemoptysis during seizure-like episodes [17]. Hemoptysis typically prompts evaluation for pulmonary, cardiovascular, hematological, infectious, and gastrointestinal disorders. In our patient, comprehensive investigations, including hematological testing, upper gastrointestinal endoscopy, chest imaging, bronchoscopy, and otorhinolaryngological assessment, failed to identify an underlying organic cause. Although pulmonary hemorrhage and diffuse alveolar hemorrhage have been reported following generalized tonic-clonic epileptic seizures, these events are uncommon and are believed to result from transient increases in pulmonary vascular pressure and permeability [18-20]. In contrast, hemoptysis associated with PNES has rarely been described in the literature. A recent case report described comparably unexplained recurrent blood-spitting in a child, occurring specifically during episodes of PNES during periods of psychosocial stress, with no organic bleeding source identified despite an extensive multidisciplinary workup where a 10-year-old boy presented with daily recurrent blood-spitting as scant blood-tinged saliva for 12 months, with episodes consistently co-occurring with psychogenic nonepileptic seizures during periods of psychosocial stress, and a comprehensive multidisciplinary evaluation revealed no organic source of bleeding [21,22]. This supports the interpretation that hemoptysis-like bleeding in the present case represents part of the same functional symptom spectrum rather than an independent organic process. The temporal association between hemoptysis and seizure-like episodes, together with the absence of identifiable structural pathology, suggests that hemoptysis in this patient represented an unusual manifestation occurring in the setting of FND.

Management of FND requires a multidisciplinary approach involving neurologists, psychiatrists, psychologists, patients, and caregivers. Effective communication of the diagnosis is a critical first step and has been shown to improve patient acceptance and engagement with treatment [21]. Psychotherapeutic interventions, particularly cognitive behavioural therapy, remain the cornerstone of management, while pharmacological treatment is reserved primarily for coexisting psychiatric conditions, such as anxiety or depression [23,24]. This case expands the clinical spectrum of FND and highlights the importance of considering functional etiologies in patients presenting with recurrent seizure-like episodes and unexplained hemoptysis after thorough exclusion of organic disease. Early recognition may reduce unnecessary investigations, prevent inappropriate treatment, and facilitate timely multidisciplinary management. Further research is needed to clarify the mechanisms underlying somatic bleeding phenomena in FND, including whether autonomic or mucosal microvascular changes contribute to pseudohemoptysis. Larger case series and prospective studies could help establish the true prevalence of this presentation, standardize diagnostic evaluation to minimize unnecessary invasive testing, and evaluate long-term outcomes of combined psychotherapeutic and pharmacological management in pediatric and adolescent populations with FND.

## Conclusions

We report a rare case of functional neurological disorder presenting as psychogenic non-epileptic seizures accompanied by recurrent hemoptysis in an adolescent patient. The coexistence of seizure-like episodes and hemoptysis posed a significant diagnostic challenge and prompted extensive neurological, pulmonary, gastrointestinal, hematological, and otorhinolaryngological evaluation, all of which failed to identify an organic cause. This case highlights the importance of considering functional neurological disorder in patients with recurrent unexplained seizure-like episodes after appropriate exclusion of underlying medical conditions. Recognition of atypical presentations of functional neurological disorder is essential to avoid unnecessary investigations and interventions. A multidisciplinary approach involving neurology, psychiatry, psychological support, patients, and caregivers can facilitate timely diagnosis, improve treatment engagement, and optimize clinical outcomes.
